# Impact of the introduction of EBUS on time to management decision, complications, and invasive modalities used to diagnose and stage lung cancer: a pragmatic pre-post study

**DOI:** 10.1186/s12885-016-2081-z

**Published:** 2016-01-28

**Authors:** Neli S. Slavova-Azmanova, Catalina Lizama, Claire E. Johnson, Herbert P. Ludewick, Leanne Lester, Shanka Karunarathne, Martin Phillips

**Affiliations:** Cancer and Palliative Care Research and Evaluation Unit (CaPCREU), School of Surgery, The University of Western Australia, Perth, 6009 WA Australia; Health Promotion Evaluation Unit, School of Sport Science, Exercise and Health, The University of Western Australia, Perth, 6009 WA Australia; Department of Respiratory Medicine, Sir Charles Gairdner Hospital, Perth, 6009 WA Australia

**Keywords:** Lung neoplasms, Diagnostic techniques and procedures, Fine needle aspiration, Bronchoscopy, EBUS, Complication

## Abstract

**Background:**

Utilisation of endobronchial ultrasound-guided transbronchial needle aspiration (EBUS-TBNA) and guide sheath (EBUS-GS) for diagnosis and staging of lung cancer is gaining popularity, however, its impact on clinical practice is unclear. This study aimed to determine the impact of the introduction of endobronchial ultrasound-guided procedures (EBUS) on time to management decision for lung cancer patients, and on the utilisation of other invasive diagnostic modalities, including CT-guided trans-thoracic needle aspiration (CT-TTNA), bronchoscopy, and mediastinoscopy.

**Methods:**

Hospital records of new primary lung cancer patients presenting in 2007 and 2008 (Pre-EBUS cohort) and in 2010 and 2011 (Post-EBUS cohort) were reviewed retrospectively.

**Results:**

The Pre-EBUS cohort included 234 patients. Of the 326 patients in the Post-EBUS cohort, 90 had an EBUS procedure (EBUS-TBNA for 19.0 % and EBUS-GS for 10.4 % of cases). The number of CT-TTNAs and bronchoscopies decreased following the introduction of EBUS (*p* = 0.015 and *p* < 0.001 respectively). Of 162 CT-TTNAs, 59 (36 %) resulted in complications compared to 1 complication each for bronchoscopy and EBUS-GS, and no complications from EBUS-TBNA. Fewer complications occurred overall in the Post-EBUS cohort compared to the Pre-EBUS cohort (*p* = 0.0264). The median time to management decision was 17 days (IQR 24) for the Pre-EBUS and 13 days (IQR 21) for the Post-EBUS cohort (*p* = 0.07). Within the Post-EBUS cohort, median time to management decision was longer for the EBUS group (*n* = 90) than the Non-EBUS group (17 days (IQR 29) vs. 10 days (IQR 10), *p* < 0.001). For half of EBUS-TBNA patients (*n* = 28, 50.0 %) and EBUS-GS patients (*n* = 14, 50.0 %), EBUS alone provided sufficient diagnostic and/or staging information; these patients had median time to management decision of 10 days. Regression analysis revealed that the number of imaging events, inpatient, and outpatient visits were significant predictors of time to management decision of >28 days; EBUS was not a predictor of time to management decision.

**Conclusions:**

The introduction of EBUS led to fewer CT-TTNAs and bronchoscopies and did not impact on the time to management decision. EBUS-TBNA or EBUS-GS alone provided sufficient information for diagnosis and/or regional staging in half of the lung cancer patients referred for this investigation.

## Background

The management of lung cancer has changed considerably over the last 5 to 10 years, with the recognition that Non-Small Cell Lung Cancer (NSCLC) is a heterogeneous disease in terms of its histopathology, molecular pathology, clinical manifestation, and response to treatment [1, 2]. Chemotherapeutic regimens are now tailored to the histological phenotype and targeted therapies are available for certain molecular pathologies [2, 3]. Consequently, tissue is required for accurate characterisation of the tumour and staging remains important for determining the appropriate treatment and for guiding prognosis.

Whilst non-invasive procedures such as computed tomography (CT), positron emission tomography (PET), and PET-CT provide information about extra-thoracic spread of tumours, their sensitivity and specificity for staging localised and regional disease such as hilar or mediastinal lymph node involvement is relatively poor [4–7]. Mediastinoscopy has been the gold standard for determining mediastinal lymph node status, but is variably performed [7, 8]. Conventional or ‘blind’ transbronchial needle aspiration (TBNA) of hilar and mediastinal lymph nodes gives inconsistent results and has not been routinely conducted [9].

The more recent advent of ultrasound-guided endoscopic procedures provides visualisation of structures on the outside of the lumen wall, thereby allowing more accurate sampling of tissue. Endobronchial ultrasound (EBUS) and oesophageal ultrasound (EUS) procedures utilise a linear probe which provides a fan-shaped ultrasound image in which the sampling needle can be seen in real time, thus allowing more accurate sampling of mediastinal and hilar lymph nodes. These procedures perform at least as well as mediastinoscopy [10]. EBUS transbronchial needle aspiration (EBUS-TBNA) - known as linear EBUS - also has the potential to sample lymph nodes at the hilum that are inaccessible to mediastinoscopy.

Over the last several decades, there has been a shift in the histology of NSCLC from squamous cell carcinoma, which tends to involve more central airways, to adenocarcinoma that is often located in the lung periphery, where approximately 70 % of NSCLC is now found [11]. In the past, sampling of such lesions was done by standard bronchoscopy with fluoroscopic guidance, which has a poor yield [12, 13]; CT guided transthoracic needle aspiration (CT-TTNA), which has a better yield but may result in complications such as pneumothorax [14]; or surgical resection, which carries some morbidity. Bronchoscopy using a radial ultrasound probe with guide sheath (EBUS-GS)–known as radial EBUS—has the potential to provide a similar diagnostic yield to CT-TTNA but with fewer complications such as pneumothorax [14].

Studies into the modalities used to diagnose lung cancer have shown a reduction in the number of CT-TTNAs following the introduction of EBUS-GS [14] and a reduction in the number of mediastinoscopies and bronchoscopies following the introduction of EBUS-TBNA [15]. However, to our knowledge, no study has simultaneously explored the impact of EBUS on all diagnostic procedures undertaken, complications arising from the various modalities, and changes to time taken from first presentation to diagnosis following the introduction of EBUS.

This study aimed to compare the number and type of procedures undertaken to diagnose and stage lung cancer, the time between first presentation at the hospital and establishment of a management decision, and the incidence of complications arising from diagnostic procedures before and after the introduction of EBUS.

## Methods

We conducted a retrospective pre-post study of all new primary lung cancer cases presented to the lung cancer Multi-Disciplinary Team Meeting (MDM) at a tertiary hospital in Western Australia, between 1 January 2007 and 31 December 2008 (Pre-EBUS cohort) and between 1 January 2010 and 31 December 2011 (Post-EBUS cohort). EBUS was introduced at the hospital at the end of 2008 and this hospital was the only site in the state where EBUS procedures were performed at the time. Patients’ medical records and hospital data were reviewed. Patients were excluded if their case was not discussed at the lung cancer MDM. While cases with both initial investigation and treatment performed outside the hospital were excluded, patients were included if they had had some imaging and/or invasive procedures performed elsewhere but were presented to the lung cancer MDM for diagnosis and management.

The following data were collected: demographic details; co-morbidities (Charlson Index) [16]; performance status (Eastern Co-operative Oncology Group Performance Status (ECOG-PS)) [17]; date of first presentation at the hospital; invasive diagnostic procedures including bronchoscopies (bronchoscopy refers to flexible bronchoscopy with bronchial brushing, washing, biopsies, and/or “blind” TBNA), CT-TTNA, EBUS, mediastinoscopy; ultrasound-guided-FNA; endoscopic ultrasound-guided-fine needle aspirations (EUS-FNA); date of procedures and resulting complications; stage of cancer; date of initial treatment decision; and date of MDM discussion(s). In addition, all occasions of services related to the lung cancer diagnosis were recorded, such as radiology/imaging investigations, outpatient visits, day case visits, inpatient visits, and visits to the accident and emergency department.

Clinical stage of the Pre-EBUS cohort was based on the 6^th^ edition of TNM staging [18], while the stage of the Post-EBUS cohort was based on the 7^th^ edition [19]. When staging was not available, clinical stage was determined from hospital data and review of imaging by a respiratory physician or respiratory fellow (authors MP and SK). Cases without histological confirmation of their lung cancer diagnosis (where diagnosis was based on imaging and clinical presentation) were allocated to the NSCLC subgroup for the purpose of analysis.

In most cases, patients were presented to our MDM after an initial CT of the thorax and upper abdomen, and in the majority of the cases results of a PET scan guided recommendations for an EBUS-TBNA investigation.

EBUS procedures: Both EBUS-TBNA and -GS investigations were performed under general anaesthesia or moderate sedation. An on-site pathologist was present to provide rapid on-site evaluation (ROSE) on EBUS-TBNA procedures. The site and number of lymph node stations sampled and the number of passes per lymph node were determined by the operator. At least three needle passes were made per lymph node unless the diagnostic material was reported adequate on ROSE.

### Statistical analysis

All statistical analyses were undertaken using IBM SPSS Statistics 19 and STATA v 13. Pearson’s chi-squared analyses or Fisher’s exact tests were undertaken for between-group comparisons for categorical variables (differences in gender, smoking status, remoteness, tumour type, and surgery between Pre-EBUS and Post-EBUS cohorts and within the Post-EBUS cohort, the EBUS and non-EBUS groups and for time to management decision (TMD) <28 days for the Post-EBUS cohort and within the EBUS group). Medians were calculated for continuous variables and non-parametric tests (Mann–Whitney U tests) were undertaken to compare groups (differences in age between Pre-EBUS and Post-EBUS cohorts and within the Post-EBUS cohort and the EBUS and non-EBUS groups; differences in the number of invasive diagnostic procedures, total number of occasions of services, and time to management decisions for the Post-EBUS cohort and within the EBUS group; and time to management decision within EBUS-GS and EBUS-TBNA). Backwards stepwise logistic regression was used to determine significant predictors of the TMD within 28 days vs greater than 28 days, with demographic variables (age, gender, remoteness), referral source, Charlson index, ECOG-PS, EBUS procedure, number of other invasive procedures, number of inpatient and outpatient visits, number of imaging investigations and stage of cancer initially entered into the model as potential predictors.

Date of first presentation at the hospital was considered to be the first lung cancer-related hospital presentation date as either an inpatient or an outpatient. Date of management decision was defined as the date of the lung cancer MDM when the diagnosis was established and/or the initial treatment decision was made. Time to management decision (TMD) was defined as time from first presentation at the hospital to date of MDM when management decision was made. Patients referred to our hospital for investigation of a lung mass were first seen in a fast track clinic, held once weekly. EBUS bronchoscopy sessions were approximately once weekly. Access to PET was usually within 7 to 10 days. MDMs at our institution are held on a weekly basis.

Ethics approval was obtained from the Sir Charles Gairdner Group Human Research Ethics Committee (REF No.2012-121) and the University of Western Australia Ethics Committee (REF No. RA/4/1/5871). The need for informed consent was waived by the Sir Charles Gairdner Group Human Research Ethics Committee.

## Results

**Fig. 1:**
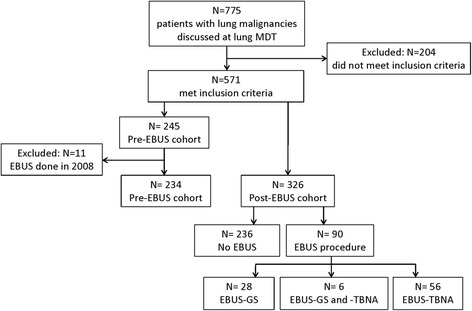
Cohort diagram of the study

Of 775 lung cancer patients presented to the lung cancer MDM, 571 met the inclusion criteria: 245 in the Pre-EBUS cohort and 326 in the Post-EBUS cohort (Fig. 1). Eleven cases in the Pre-EBUS cohort underwent EBUS and were excluded from the study as the respiratory team was learning the new technique and in some cases an additional procedure was performed to confirm the EBUS result.

### Patient characteristics

Both Pre-EBUS and Post-EBUS cohorts had similar patient characteristics (Table 1). Within the Post-EBUS cohort, no significant demographic differences were found between the patients who had an EBUS investigation (EBUS group) and those who did not (non-EBUS group) (Table 1). There were significant differences between the EBUS and non-EBUS group in terms of ECOG-PS (*p* = 0.009); EBUS was undertaken mainly for patients with better performance status (ECOG-PS of 0 and 1).

**Table 1:** Patient characteristics of both cohorts, and of EBUS and Non-EBUS patients within the Post-EBUS cohort

Patient characteristics	Both cohorts		Post-EBUS cohort (*n* = 326)	
	Pre-EBUS cohort (*n* = 234)	Post-EBUS cohort (*n* = 326)	EBUS group (*n* = 90)	Non-EBUS group (*n* = 236)
	Median (IQR)	Median (IQR)	Median (IQR)	Median (IQR)
Age at diagnosis (years)^a^	69 (15)	69 (17)	67 (15)	70 (18)
	n (%)	n (%)	n (%)	n (%)
Male	139 (59.4)	200 (61.3)	58 (64.4)	142 (60.2)
Smoker				
Unknown	18 (7.7)	32 (9.8)	8 (8.9)	24 (10.2)
Current	77 (32.9)	92 (28.2)	29 (32.2)	63 (26.7)
Ceased	124 (53.0)	180 (55.2)	49 (54.4)	131 (55.5)
Never	15 (6.4)	22 (6.7)	4 (4.4)	18 (7.6)
Remoteness				
Major city	184 (79.3)	244 (74.8)	65 (72.2)	179 (75.8)
Inner regional	17 (7.3)	29 (8.9)	10 (11.1)	19 (8.1)
Outer regional	22 (9.5)	34 (10.4)	11 (12.2)	23 (9.7)
Remote	9 (3.9)	19 (5.8)	4 (4.4)	15 (6.4)
ECOG-PS^b^				
0	87 (37.2)	91 (27.9)	25 (28.0)	66 (27.8)
1	78 (33.3)	143 (43.9)	50 (55.6)	93 (39.5)
2	43 (18.4)	58 (17.8)	13 (14.4)	45 (19.1)
3	20 (8.5)	28 (8.6)	2 (2.2)	26 (11.0)
4	6 (2.6)	6 (1.8)	0 (0.0)	6 (2.0)
Tumour type				
NSCLC	204 (87.2)	288 (88.3)	80 (88.9)	208 (88.1)
SCLC	30 (12.8)	38 (11.7)	10 (11.0)	28 (11.9)

^a^Mann–Whitney U test; all others except ^b^are Pearson’s chi squared

^b^No significant differences between groups except for ECOG-PS (EBUS group compared with Non-EBUS group, Fisher’s exact test, *p* = 0.009)

### Invasive procedures

The main invasive procedures in the Pre-EBUS cohort were bronchoscopy and CT-TTNA, and in the Post-EBUS cohort, bronchoscopy, CT-TTNA, and EBUS (Table 2). There was a 17.5 % reduction in the proportion of patients who had bronchoscopies (*p* < 0.001) and a 10.2 % fall in the proportion of patients with CT-TTNA (*p* = 0.012) following the introduction of EBUS-TBNA and EBUS-GS. Mediastinoscopies were not routinely performed on lung cancer patients in either cohort; only one mediastinoscopy was performed in the Pre-EBUS cohort and three in the Post-EBUS cohort. In the Post-EBUS cohort, EBUS-TBNA was undertaken for 19.0 % of cases (*n* = 62) and EBUS-GS for 10.4 % of cases (*n* = 34) (Table 3). EBUS-GS was utilised equally across stages for NSCLC patients, but not used for any SCLC patients. EBUS-TBNA was utilised equally across both NSCLC and SCLC patients, with a greater proportion of Stage III NSCLC (30.7 %) and Limited SCLC (44.4 %) patients undergoing EBUS-TBNA than other stages.

**Table 2:** Patients receiving invasive procedures, time to management decision, and diagnostic procedures for the Pre-EBUS cohort compared to the Post-EBUS cohort

	Pre-EBUS cohort (*n* = 234)	Post-EBUS cohort (n = 326)	p
	n (%)^a^	n (%)^a^	
Invasive procedures			
Bronchoscopy	135 (57.7)	131 (40.2)	<0.001**
CT-TTNA	92 (39.3)	95 (29.1)	0.012*
EBUS	0 (0)	90 (27.6)	<0.001**
Thoracentesis	26 (11.1)	24 (7.4)	0.125
Other invasive procedures	30 (12.8)	47 (14.4)	0.588
Other surgical procedures	9 (3.8)	13 (4.0)	0.932
Mediastinoscopy	1 (0.4)	3 (0.9)	0.644^d^
Time to management decision^c^			
≤ 28 days	154 (66.7)	245 (75.9)	
> 28 days	77 (33.3)	78 (24.1)	0.018*
	Median (IQR)	Median (IQR)	
Time to management decision (days)^c^	17 (24)	13 (21)	0.070^d^
Number of invasive diagnostic procedures^b^	1 (0)	1 (0)	0.842^e^

Thoracentesis: thoracentesis, pleural effusion drainage, pleural biopsy

Other invasive procedures: FNA, US-FNA, EUS-FNA, biopsy other, CT biopsy other

Other surgical therapeutic/diagnostic procedures: surgery for brain metastasis, bone marrow trephine, spinal lesions

*p < 0.05, **p < 0.01

^a^Number of patients

^b^Number per patient

^c^Excludes 6 patients with no date of presentation available – unable to establish time to management decision

^d^Fisher’s exact test

^e^Mann–Whitney U test; all other tests except ^d^ are Pearson’s chi squared test

**Table 3:** Number and stage of patients in the Post-EBUS cohort (*N* = 326) who had EBUS

	EBUS-GS group	EBUS-TBNA group
Stage (n)	n (%^a^)	n (%^a^)
NSCLC		
I (55)	9 (16.4)	6 (10.9)
II (23)	4 (17.4)	2 (8.7)
III (75)	9 (12.0)	23 (30.7)
IV (135)	12 (8.9)	21 (15.6)
SCLC		
Limited (18)	0 (0)	8 (44.4)
Extensive (20)	0 (0)	2 (10.0)
Overall (326)	34 (10.4)^b^	62 (19.0)^b^

^a^Percentage within cancer stage

^b^Includes 6 patients who had both EBUS-GS and EBUS-TBNA

### Number of invasive procedures per patient

A median of one invasive procedure was performed per patient in both cohorts (Table 2) (*p* = 0.842). One invasive procedure was sufficient to establish lung cancer diagnosis for 68 % of patients in both cohorts. Invasive procedures were not undertaken on 8.5 % of the patients in the Pre-EBUS cohort and 9.8 % in the Post-EBUS cohort, for whom a diagnosis of lung cancer was made on the basis of clinical presentation and imaging. Approximately 23 % of the patients in the Pre-EBUS cohort and 22 % of the Post-EBUS cohort had 2 or more invasive procedures.

Six patients underwent both EBUS-GS and EBUS-TBNA; five as part of a single procedure and in one case EBUS-GS was undertaken with diagnostic purpose and then followed up by EBUS-TBNA for staging. A single EBUS-GS investigation was sufficient to establish lung cancer diagnosis in 41.2 % (*n* = 14) of all EBUS-GS cases. One patient (2.9 %) had two EBUS-GS procedures, nine patients (26.5 %) underwent other invasive investigations following EBUS-GS, and four patients (11.8 %) had invasive procedures before EBUS-GS. The main reason for additional invasive investigations among EBUS-GS patients was inadequacy of the preceding investigation/s. One patient was referred for EBUS-CG for material for molecular testing following positive result from a bronchoscopy.

Approximately half of all patients undergoing EBUS-TBNA (45.2 %, *n* = 28) also underwent additional invasive investigations, both prior to and following the EBUS procedure. Multiple procedures were required for a number of reasons, including: non-diagnostic results from initial invasive investigations (19.4 %, *n* = 12); non-diagnostic EBUS-TBNA results (9.7 %, *n* = 6); additional material required for molecular testing (1.6 %, *n* = 1); and EBUS-TBNA being conducted for staging purposes only, following positive diagnosis from CT-TTNAs and FBs (12.9 %, *n* = 8).

### Complications

Across both cohorts, 36 % of CT-TTNAs resulted in complications. Of 162 CT-TTNAs, 57 resulted in a pneumothorax, one in pulmonary haemorrhage, and one in intra-parenchymal bleeding. While only nine cases with pneumothorax following CT-TTNA had chest tube inserted, 32 patients were admitted for observation overnight. Only one complication (a small pneumothorax) occurred each as a result of bronchoscopy (*N* = 260) and EBUS-GS (*N* = 34). EBUS-TBNA (*N* = 62) did not result in any complications. Significantly fewer complications occurred in the Post-EBUS cohort compared to the Pre-EBUS cohort (9.0 % vs 15.3 %; *χ*^*2*^ = 4.931; *p* = 0.0264).

### Time to management decision

The median TMD was 17 days for the Pre-EBUS cohort and 13 days for the Post-EBUS cohort (*p* = 0.070) (Table 2). In the Post-EBUS cohort, when EBUS was the only invasive procedure undertaken, the median TMD was comparable to the non-EBUS patients: 10 days for both EBUS-GS and EBUS-TBNA (Table 4). However, half of EBUS-TBNA patients (*n* = 28, 50.0 %) and EBUS-GS patients (*n* = 14, 50.0 %) underwent EBUS before or after other invasive investigations. For these patients, median TMD was longer compared to patients with EBUS only: 45 days for EBUS-GS (*p* = 0.001) and 26.5 days for EBUS-TBNA (*p* < 0.001) (Table 4). More patients in the Post-EBUS cohort were diagnosed within 28 days of presenting at the hospital when compared to the Pre-EBUS cohort (75.9 % vs 66.7 %; *p* = 0.018) (Table 2).

**Table 4:** Time to management decision for patients with EBUS as the only invasive investigation compared to patients with EBUS combined with other invasive investigations (Post-EBUS cohort *N* = 84^a^)

	Single EBUS-GS only (*n* = 14) median (IQR)	EBUS-GS plus other invasive investigations (*n* = 14)^b^median (IQR)	p^c^	Single EBUS-TBNA only (*n* = 28) median (IQR)	EBUS-TBNA plus other invasive investigations (*n* = 28) median (IQR)	p^c^
Time to management decision (days)	10 (28)	45 (48)	0.001*	10 (10.0)	26.5 (29)	<0.001*

*p < 0.01

^a^excludes 6 patients with both EBUS-GS and EBUS-TBNA

^b^includes 1 patient with 2 EBUS-GS investigations

^c^Mann–Whitney U test

A multiple logistic regression identified predictors of TMD within 28 days. The total number of inpatient visits, outpatient visits and imaging investigations (Table 5) predicted a longer TMD. Thus, reduced odds of TMD of less than 28 days occurred with higher numbers of inpatient visits (OR = 0.64, *p* = 0.020), outpatient visits (OR = 0.37, *p* < 0.001) or imaging investigations (OR = 0.81, *p* < 0.001). Conversely, patients with Stage III (OR = 3.16, *p* = 0.002) or Stage IV (OR = 4.73, p < 0.001) NSCLC had increased odds of TMD within 28 days compared to those with Stage I NSCLC. Patients with Limited (OR = 5.93, *p* = 0.011) or Extensive (OR = 4.64, *p* = 0.009) SCLC had increased odds of TMD within 28 days compared to those with Stage I NSCLC. EBUS was not an independent predictor of TMD within 28 days.

**Table 5:** Logistic regression predictors of time to management decision

	OR	95 % LCI	95 % UCI	p
EBUS	1.29	0.60	2.75	0.516
Total number of inpatient visits	0.64	0.44	0.93	0.020*
Total number of outpatient visits	0.37	0.29	0.48	<0.001**
Number of invasive procedures	0.65	0.39	1.08	0.096
Total number of imaging investigations	0.81	0.72	0.91	<0.001**
NSCLC				
Stage II	2.22	0.79	6.27	0.130
Stage III	3.16	1.54	6.52	0.002**
Stage IV	4.73	2.39	9.36	<0.001**
SCLC				
Limited	5.93	1.51	23.27	0.011*
Extensive	4.64	1.48	14.61	0.009**

Comparisons: Time to diagnosis less than or equal to 28 days vs greater than 28 days, EBUS compared to non-EBUS, Stage compared to NSCLC Stage I

*p < 0.05, **p < 0.01

To assess change in practice patterns between the Pre- and Post-EBUS cohort, we evaluated all NSCLC patients with Stage I, II and III disease who had surgical resection (Table 6). A higher proportion of patients with clinical Stage II (N1 involvement) in the Post-EBUS cohort proceeded to surgery, compared with the Pre-EBUS cohort.

**Table 6:** NSCLC patients (stage I, II and III) with surgical resection in the Pre-EBUS cohort compared to the Post-EBUS cohort

	Pre-EBUS cohort (*n* = 97)		Post-EBUS cohort (*n* = 153)		
NSCLC stage	Surgery n (%)	No surgery n (%)	Surgery n (%)	No surgery n (%)	p
I	16 (59.3)	11 (40.7)	36 (65.5)	19 (34.5)	0.378^a^
II	2 (16.7)	10 (83.3)	13 (56.5)	10 (43.5)	0.026^a,^*
III	5 (8.6)	53 (91.4)	3 (4.0)	72 (96.0)	0.228^b^
Total	23 (23.7)	74 (76.3)	52 (34)	101 (66)	

*p < 0.05

^a^Pearson’s chi squared test

^b^Fisher’s exact test

## Discussion

The introduction of new diagnostic procedures has the potential to prolong the diagnostic process and contribute to a delay in management decisions. However, this retrospective pre/post study demonstrated that introduction of EBUS-GS and EBUS-TBNA for the diagnosis of lung cancer at a tertiary teaching hospital in Western Australia led to a decrease in the number of bronchoscopies and CT-TTNAs and did not affect the TMD. The Post-EBUS cohort had fewer complications, which may be attributed to the decrease in the number of CT-TTNAs, as no change in the proportion of complications resulting from CT-TTNAs was observed.

In our study, only one mediastinoscopy was performed in the Pre-EBUS cohort and three in the Post-EBUS cohort. Mediastinoscopies were not routinely performed as frequently as guidelines and their “gold standard” status would recommend [7, 8, 20]; perhaps over-reliance was placed on CT and PET scans for staging of the mediastinum. In our study, all three patients who underwent mediastinoscopies in the Post-EBUS cohort had an EBUS investigation prior to the mediastinoscopy and mediastinoscopies were the last investigation in a rigorous workup required to determine precise stage of the disease and suitability for surgical treatment.

Our results show that diagnosis and staging of lung cancer in the Pre-EBUS cohort was a two stage process, with sampling of the peripheral lung mass by CT-TTNA or FB, and staging of the mediastinum by PET scanning, despite its limitations. Whilst mediastinoscopy was rarely performed at our hospital, previous studies of patients with NSCLC have reported that evaluation of the mediastinum by mediastinoscopy was infrequently performed (27 %) in patients undergoing surgery [7, 8]. In addition, a recent multicentre, pragmatic, randomised controlled trial (RCT) substantiated that mediastinoscopy is rarely needed for the pre-operative staging of NSCLC in clinical practice [21]. A prospective clinical trial by Navani et al. [22] suggests that EBUS-TBNA may prevent 87 % of mediastinoscopies if routinely performed for patients with mediastinal lymph node involvement.

With regard to EBUS-TBNA staging of N2 nodes, there were no false negative cases discovered at surgery. A higher proportion of patients with clinical Stage II (N1 involvement) in the Post-EBUS cohort proceeded to surgery, compared with the Pre-EBUS cohort (Table 6). Following the introduction of EBUS, there was an increase in patients being considered for surgery; PET scans are known to be oversensitive and thus may have unnecessarily excluded some patients in the Pre-EBUS cohort from having surgery.

Surgery is indicated for patients with Stage I or II disease and good performance status; hence, accurate staging is essential to exclude mediastinal involvement. Patients with an ECOG-PS of 3 or 4 are less suitable for radical treatment and thus fewer patients would be referred for EBUS-TBNA, as mediastinal staging is less critical. Consistent with these recommendations, almost all EBUS patients in our study had ECOG-PS of 0, 1, or 2.

Radical chemo-radiotherapy with curative intent is indicated for Stage III disease. Our findings show that a higher proportion of Stage III NSCLC cases underwent EBUS-TBNA when compared to the other stages. As these patients are more likely to present with enlarged lymph nodes on CT or PET imaging, EBUS-TBNA would be the preferred invasive procedure, providing both diagnostic and staging information simultaneously with a lower risk of complication. EBUS-TBNA was undertaken in almost half of the patients with limited SCLC; such cases often have enlarged hilar or mediastinal lymph nodes, so EBUS-TBNA provides diagnostic material.

Recommended timelines for diagnosis and start of treatment for lung cancer have been included in several guidelines [23, 24] and are considered to be indicators of quality of health-care. Whilst there is no established relationship between time to diagnosis or treatment and survival/recurrence in lung cancer patients, delays may contribute to distress in patients and missed opportunities to treat [25, 26]. Currently, Western Australian guidelines recommend four weeks (28 days) from initial presentation to specialist to initial treatment decision [27].

This study demonstrated that the median time from initial presentation to management decision for lung cancer patients decreased from 17 days for the Pre-EBUS cohort to 13 days for the Post-EBUS cohort; however, this difference was not statistically significant. It is important to emphasise that patients with suspected lung cancer presented at the hospital via different pathways, although referral source was not a predictor for the time taken from first presentation to final diagnosis and treatment recommendations. A small number of patients with suspicious pulmonary lesions on imaging, required extensive work-up and long follow-up before a definitive diagnosis was established. This may be reflected in the regression analysis, which indicated that a greater number of imaging investigations and inpatient and outpatient visits were associated with TMD >28 days. Furthermore, patients with Stage III and Stage IV NSCLC and patients with SCLC had higher odds of TMD of ≤28 days when compared to Stage I NSCLC. This finding is consistent with the overall clinical management of patients with advanced lung cancer, who are less likely to be suitable for radical treatment and require less rigorous investigations to guide management decisions, and hence, take less time to decide on a management plan.

A recent multicentre, pragmatic, RCT showed that routine use of EBUS-TBNA after a staging CT for suspected lung cancer resulted in faster management decisions with fewer investigations when compared with conventional diagnosis and staging methods [21]. Our study found that EBUS was not an independent predictor of shorter TMD when introduced into routine clinical practice at a tertiary hospital providing a state-wide service. However, median TMD in our pre-EBUS cohort was 17 days, substantially less than the 29 days reported by Navani et al. for patients receiving conventional diagnosis and staging [21].

Our results also showed that in the Post-EBUS cohort, for patients who had only a single EBUS (TBNA or GS) investigation, median TMD was comparable to those who had conventional invasive diagnostic and staging investigations. EBUS-TBNA alone provided diagnosis and intra-thoracic regional staging in 50 % of cases and a single EBUS-GS investigation was sufficient to establish lung cancer diagnosis in 50 % of cases undergoing the respective procedure.

For half of the EBUS cases, additional invasive diagnostic procedures were conducted to obtain a definitive diagnosis and/or staging; these patients had a longer TMD. This may be explained by the finding that greater numbers of imaging procedures and occasions of service were both predictors of increased TMD in the regression analysis, and points toward the potential complexity of some cases referred for EBUS.

While EBUS-GS provides diagnostic information only, EBUS-TBNA may fulfil both diagnostic and staging purposes, particularly in patients with suspected mediastinal lymph node involvement, where evidence-based guidelines recommend sampling of the mediastinum as the most appropriate first invasive test [20]. Multiple procedures were required for a number of reasons, including: non-diagnostic results from initial invasive investigations; additional material required for molecular testing and concerns about extra-thoracic disease. Nine patients in the second cohort had lung cancer diagnosis confirmed with either CT-TTNA, FB or EBUS-GS but following this, also underwent EBUS-TBNA. In these cases, the EBUS-TBNA procedure was performed for the purposes of staging, where prior to the introduction of EBUS, this should have been confirmed with mediastinoscopy. Given that the mediastinoscopies were underutilised at our hospital, such cases presenting in the first cohort would most likely have been staged via PET alone; therefore, while the availability of EBUS may have led to additional procedures being performed in these cases (and a subsequent delay in TMD), the advantage of more accurate staging must be recognised.

In this study, EBUS procedures were undertaken in 28 % of newly diagnosed lung cancer patients, with EBUS more likely to be undertaken in diagnostically challenging cases. As experience with EBUS has developed, there has subsequently been an increase in its use, such that EBUS is now performed in approximately 74 % of lung cancer patients at the hospital (unpublished results) for the purposes of tissue acquisition and more accurate staging.

We recognise that our study has several limitations. This was a single-centre retrospective study in one of the largest tertiary hospitals in Western Australia that services a diverse population of patients, some of whom were referred from long distances and private practices because it was the only site in Western Australia to perform EBUS at the time. As such, our study cohort may not be representative of other practice. However, the diversity of referrals and the large geographic catchment area of the patients included in the study support our assumption that local variations are less likely to contribute to the reported findings, and that our results may be generalisable to other institutions providing similar care. Some may argue that the small number of mediastinoscopies performed over both cohorts limits the study’s generalisability. While we agree that mediastinoscopy has traditionally been considered the “gold standard” for lymph node sampling in patients with suspected lung cancer and mediastinal adenopathy, previous studies have reported that mediastinoscopy has been widely underused [7, 8] and more recent findings indicate that mediastinoscopy is rarely needed for preoperative staging of NSCLC in clinical practice. Furthermore, the latest guidelines from the American College of Chest Physicians recommend EBUS-TBNA as a primary invasive investigation over surgical staging in lung cancer patients with suspected mediastinal lymph node involvement [20]. It should be, however, recognised that the guidelines also recommend that surgical staging be considered in cases where the clinical suspicion of mediastinal node involvement remains high after a negative result using a needle technique.

## Conclusions

Our study shows that the introduction of EBUS to diagnose lung cancer was associated with a reduction in CT-TTNAs, bronchoscopies, and complications resulting from the invasive procedures. Furthermore, the institution of EBUS did not extend TMD, which remains well within current guidelines. In addition, EBUS alone provided sufficient diagnostic and/or regional staging information in 50 % of both EBUS-TBNA and EBUS-GS cases.

## Abbreviations

CT: computed tomography
CT-TTNA: CT-guided trans-thoracic needle aspiration
EBUS: endobronchial ultrasound
EBUS-GS: endobronchial ultrasound guide sheath
EBUS-TBNA: endobronchial ultrasound-guided transbronchial needle aspiration
ECOG-PS: Eastern Co-operative Oncology Group Performance Status
EUS: oesophageal ultrasound
EUS-FNA: endoscopic ultrasound-guided fine needle aspiration
FNA: fine needle aspiration
MDM: multi-disciplinary team meeting
NSCLC: non-small cell lung cancer
PET: positron emission tomography
SCLC: small cell lung cancer
TBNA: transbronchial needle aspiration
TMD: time to management decision
